# Mitochondrial Targeting in an Anti-Austerity Approach Involving Bioactive Metabolites Isolated from the Marine-Derived Fungus *Aspergillus* sp.

**DOI:** 10.3390/md18110555

**Published:** 2020-11-07

**Authors:** Waleed A Abdel-Naime, Atsushi Kimishima, Andi Setiawan, John Refaat Fahim, Mostafa A. Fouad, Mohamed Salah Kamel, Masayoshi Arai

**Affiliations:** 1 Graduate School of Pharmaceutical Sciences, Osaka University, 1-6 Yamadaoka, Suita, Osaka 565-0871, Japan; waleed_cognosy@yahoo.com (W.A.A.-N.); kimishima-a@phs.osaka-u.ac.jp (A.K.); 2 Department of Pharmacognosy, Faculty of Pharmacy, Minia University, Minia 61519, Egypt; Johnrefaat82@yahoo.com (J.R.F.); m_fouad2000@yahoo.com (M.A.F.); 3 Department of Chemistry, Faculty of Science, Lampung University, J1. Prof. Dr. Sumantri Brodjonegoro No. 1, Bandar Lampung 35145, Indonesia; andi.setiawan@fmipa.unila.ac.id; 4 Department of Pharmacognosy, Faculty of Pharmacy, Deraya University, Universities Zone, New Minia 61111, Egypt

**Keywords:** marine-derived *Aspergillus* sp., cancer, microenvironment, nutrient starvation, austerity, physcion, mitochondrial electron transport chain

## Abstract

The tumor microenvironment is a nutrient-deficient region that alters the cancer cell phenotype to aggravate cancer pathology. The ability of cancer cells to tolerate nutrient starvation is referred to as austerity. Compounds that preferentially target cancer cells growing under nutrient-deficient conditions are being employed in anti-austerity approaches in anticancer drug discovery. Therefore, in this study, we investigated physcion (**1**) and 2-(2′,3-epoxy-1′,3′,5′-heptatrienyl)-6-hydroxy-5-(3-methyl-2-butenyl) benzaldehyde (**2**) obtained from a culture extract of the marine-derived fungus *Aspergillus* species (sp.), which were isolated from an unidentified marine sponge, as anti-austerity agents. The chemical structures of **1** and **2** were determined via spectroscopic analysis and comparison with authentic spectral data. Compounds **1** and **2** exhibited selective cytotoxicity against human pancreatic carcinoma PANC-1 cells cultured under glucose-deficient conditions, with IC_50_ values of 6.0 and 1.7 µM, respectively. Compound **2** showed higher selective growth-inhibitory activity (505-fold higher) under glucose-deficient conditions than under general culture conditions. Further analysis of the mechanism underlying the anti-austerity activity of compounds **1** and **2** against glucose-starved PANC-1 cells suggested that they inhibited the mitochondrial electron transport chain.

## 1. Introduction

Solid tumors contain nutrient-starved regions because of abnormal cell proliferation coupled with defective and disorganized vascular supply [1]. Cancer cells that have adapted to this tumor microenvironment are assumed to develop aggressive phenotypes, promoted angiogenesis, and drug resistance [2,3]. In particular, pancreatic tumors are hypovascular, leading to nutrient starvation within the tumor microenvironment [4,5]. In contrast to other cell lines, pancreatic cancer cell lines show extraordinary survival capacity under nutrient-deprived conditions [6]. In addition, pancreatic cancer is one of the most aggressive human malignancies, because it is often initially asymptomatic, so most patients already have metastases upon presentation. Furthermore, the available chemotherapeutic approaches have significant adverse effects and only marginal therapeutic efficacy.

The ability of cancer cells to tolerate starvation is referred to as austerity [7]. The nutrient-starved environments in tumors are unlike those in normal tissues. The use of the anti-austerity approach in anticancer drug discovery involves compounds that can preferentially affect cancer cells under nutrient-starvation conditions [7]. To date, some natural products, such as kigamicin D (polycyclic xanthone derivative) and ancistrolikokine E3 (naphthyldihydroisoquinoline alkaloid) isolated from the culture extract of *Amycolatopsis* sp. and the plant *Ancistrocladus likoko*, respectively, have shown selective anti-austerity effects on nutrient-starved human pancreatic carcinoma PANC-1 cells [8,9,10]. Hundreds of new compounds are identified from marine medicinal resources [11]. Accordingly, the chemical and biological diversity from marine resources acts as a target for drug discovery and potentiates us to develop methods for the detection of their relevant therapeutic goal [12]. We recently isolated DC1149B (epidithiodiketopiperazine), *N*-methylniphatin A (a new 3-alkyl pyridine alkaloid), and biakamides (unique new polyketides) from the marine-derived fungus *Trichoderma lixii*, a marine sponge of *Xestospongia* sp., and a marine sponge of *Petrosaspongia* sp., respectively; these compounds showed cytotoxic activity against PANC-1 cells that were adapted to glucose-deficient conditions [11,12,13]. An analysis of their mode of action indicated that almost all these compounds inhibited mitochondrial function [13,14,15]. Known inhibitors of mitochondrial function such as antimycin A, rotenone, and secalonic acid D have been reported to show anti-austerity activity against pancreatic cancer cells [16,17]. Furthermore, the anti-austerity agents IACS-10759 (synthetic inhibitor of complex I) and arctigenin (originally isolated from the plant *Arctium lappa*) are currently in clinical trials as anticancer drugs [18,19].

A preponderance of new evidence shows that pancreatic cancer cells are especially dependent on mitochondrial oxidative phosphorylation under low nutrient conditions and that mitochondrial metabolism represents a key metabolic vulnerability [20,21]. An siRNA screen pertaining to 2752 metabolic genes revealed that mitochondrial genes encoding components of the electron transport chain were functionally the most important genes involved in cancer cell survival under glucose-deficient conditions [22]. Therefore, new anti-austerity agents that target the mitochondrial function in pancreatic cancer cells would comprise an important class of chemotherapeutic agents against pancreatic cancer.

In this study, we aimed to obtain anti-austerity agents from marine resources and isolated physcion (**1**) and 2-(2′,3-epoxy-1′,3′,5′-heptatrienyl)-6-hydroxy-5-(3-methyl-2-butenyl) benzaldehyde (**2**) from the culture extract of a marine-derived *Aspergillus* sp. isolated from a marine sponge. We analyzed the cytotoxic effects of compounds **1** and **2** on nutrient-starved pancreatic cancer cells and their mechanisms of action, while focusing on the effects of the compounds on mitochondrial functions.

## 2. Results and Discussion

### 2.1. Isolation and Structural Analysis of Active Compounds ***1*** and ***2***

**Figure 1:**
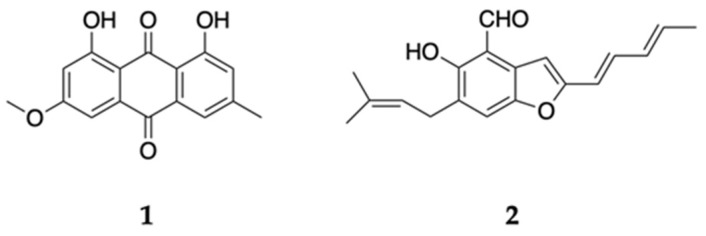
Chemical structures of isolated compounds **1** and **2**.

The marine-derived fungus *Aspergillus* sp. 18B-15-3 was isolated from an unidentified marine sponge collected on Pramuka Island, Jakarta bay, Indonesia in 2016. On the basis of the bioassay results, the 15-day-old culture extract was partitioned with water and EtOAc, and the EtOAc layer was further partitioned into a *n*-hexane–90% aqueous MeOH mixture. The *n*-hexane-soluble fraction was fractionated using silica gel open column chromatography (*n*-hexane–chloroform), normal phase high-performance liquid chromatography (HPLC), and preparative thin-layer chromatography (TLC) to obtain physcion (**1**) and 2-(2′,3-epoxy-1′,3′,5′-heptatrienyl)-6-hydroxy-5-(3-methyl-2-butenyl) benzaldehyde (**2**) (Figure 1). Compounds **1** and **2** were identified by electrospray ionization time-of-flight mass spectrometry (ESI-TOF-MS) or matrix-assisted laser desorption (MALDI)-TOF-MS and nuclear magnetic resonance (NMR) analyses and comparison with authentic spectral data [23,24,25,26] (Figures S1–S14).

### 2.2. Cytotoxic Effect of Compounds ***1*** and ***2*** on PANC-1 Cells under Glucose-Deficient and General Culture Conditions

Bioassay-guided separation of the active *n*-hexane fraction from the culture extract of the marine-derived fungus *Aspergillus* sp. 18B-15-3 let us isolate physcion (**1**) and 2-(2′,3-epoxy-1′,3′,5′-heptatrienyl)-6-hydroxy-5-(3-methyl-2-butenyl) benzaldehyde (**2**) (Figure 1). We then evaluated the cytotoxic effect of these compounds on PANC-1 cells cultured under glucose-deficient or general culture conditions. Antimycin A, which selectively shows anti-austerity effects on PANC-1 cells that have adapted to glucose-deficient conditions [16], was utilized as the positive control.

Compound **2** exhibited higher cytotoxic activity against PANC-1 cells that were adapted to glucose starvation (IC_50_ = 1.7 µM), whereas the cells cultured under general culture conditions were not affected by compound **2** (IC_50_ = 859 µM) (Table 1). The selectivity index (S.I.) based on the difference in the IC_50_ values for the general culture and glucose-deficient media for compound **2** was more than 505. Compound **1** showed a slightly weaker IC_50_ value (6.0 µM) than compound **2**. The S.I. of **1** was calculated to be 169.5. Compound **1** was previously isolated from the medicinal plants [27,28,29,30,31,32,33] and both terrestrial and marine-derived fungi, such as *Aspergillus* spp., *Cytospora eugeniae*, *Penicillium* sp., *Stemphylium lycopersici*, and *Microsporum* sp. [34,35,36,37,38]. Compound **1** is a major secondary metabolite and has been reported to exhibit antimicrobial [39,40], antiviral [41], antioxidant [42,43], and anti-inflammatory [44,45] activities. A number of studies demonstrated compound **1** had cytotoxic effects on diverse cancer cells under general culture conditions, including leukemia (U937 cell lines with IC_50_ = 27.90 µM [45], NALM6 and SUPB15 cell lines with IC_50_ = 5.00 µM [46], K562 cell lines with IC_50_ = 12.50 µM [47], CCRF-ADR5000 cells with IC_50_ = 74.79 µM [48], breast cancer cells [49] (IC_50_ = 45.4 µM), cervical carcinoma cells [38] (IC_50_ = 100 µM), colon cancer cells [50,51] (IC_50_ = 10–50 µM), lung cancer cells [47,52] (IC_50_ = 15.1–35.1 µM), nasopharyngeal carcinoma cells [53] (IC_50_ = 20 µM), and hepatocellular carcinoma cells [54,55] (IC_50_ = 10 µM), while no report was observed regarding the effects of compound **1** on the pancreatic cancer cells under both general culture and nutrient-starved conditions. The related mechanisms also have been considerably explored; compound **1** modulated the molecules or signaling transduction pathways related to apoptosis, autophagy, and the cell cycle [46,49,53,54,55]. Compound **1** also acted as a specific inhibitor of glucose-6-phosphate dehydrogenase against breast cancer cells, resulting in elevated reactive oxygen species (ROS) levels and decreased lipogenesis and RNA biosynthesis [47]. Compound **2** was previously isolated from *Aspergillus glaucus* [25] and *Eurotium* spp. [56,57,58] and evaluated for cytotoxic effects on some cancer cell lines. However, compound **2** did not show potent cytotoxicity. The IC_50_ values of compound **2** against the P-388, K-562, HL-60, and A-549 cells were found to be higher than 10 µg/mL (>35.2 µM), and the use of a higher concentration of compound **2** (100 µg/mL) (352 µM) led to more than 90% cytotoxic effect against SF-268, MCF-7, and NCI-H460 cells [58,59]. In the current study, we found that they exhibited unique biological activity—that is, anti-austerity activity. Subsequently, we investigated the mechanism underlying the effects of compounds **1** and **2.**

### 2.3. Analysis of Mechanism of Action as Anti-Austerity Agents

**Figure 2:**
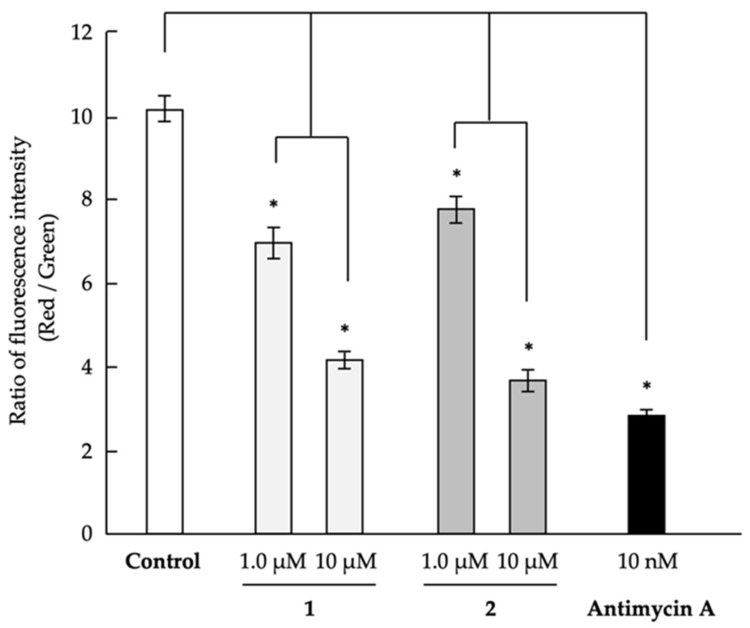
Effect of compounds **1** and **2** on the mitochondrial membrane potential of human pancreatic carcinoma PANC-1 cells. PANC-1 cells that adapted to glucose-deficient medium in the 96-well plates were treated with the indicated concentrations of the compounds for 1 h. Next, JC-1 working solution was added for measuring the mitochondrial membrane potential, and the plate was incubated for 30 min. The fluorescence intensity of the cells was measured using a Tecan Infinite M1000 microplate reader at Ex 485 nm and Em 535 nm (Green color fluorescence) and Ex 535 nm and Em 595 nm (Red color fluorescence). Differences were considered significant at * *p* < 0.05.

Antimycin A, which was used as the positive control, inhibits complex III in the mitochondrial electron transport chain [60]. In addition, other inhibitors of mitochondrial function were found to have an anti-austerity effect on cancer cells that adapted to nutrient starvation conditions [14]. Therefore, we analyzed the mitochondrial membrane potential to determine whether compounds **1** and **2** inhibited mitochondrial function (Figure 2). Both compounds were found to dose-dependently decrease mitochondrial membrane potential in PANC-1 cells, indicating that they inhibited mitochondrial function, which might be responsible for the selective cytotoxic effect of **1** and **2** on PANC-1 cells under glucose-deficient conditions. In the future, we will target the relationship between the effect of parietin or physcion with its close emodin for studying the methoxy group on the biological activity and mitochondrial effect.

**Figure 3:**
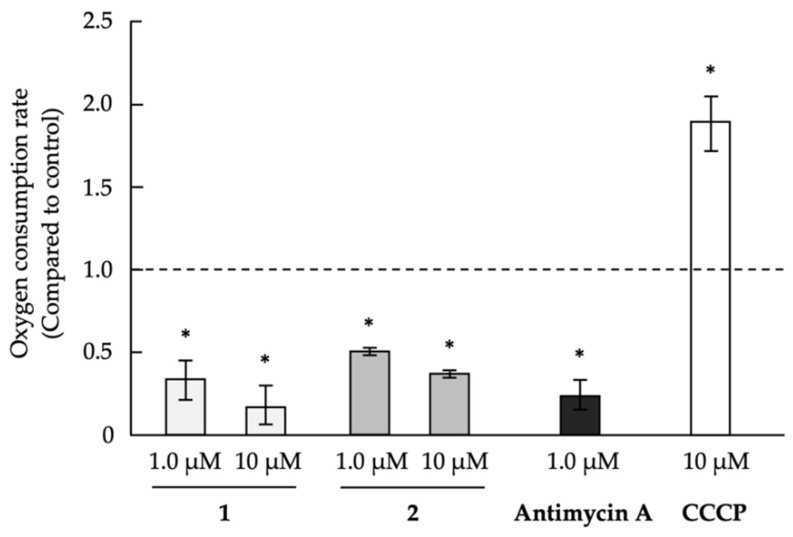
Effect of compounds **1** and **2** on the oxygen consumption by PANC-1 cells. The precultured PANC-1 cells in the 96-well plates were incubated in general culture medium for 12 h at 37 °C. The medium was then replaced with fresh medium; subsequently, the test compound was added, followed by addition of the phosphorescent probe to measure the oxygen consumption. The signals were measured with a Tecan Infinite M1000 microplate reader by using the time-resolved mode. Differences were considered significant at * *p* < 0.05. CCCP: carbonyl cyanide *m*-chlorophenylhydrazone.

We recently reported that secalonic acid D and carbonyl cyanide *m*-chlorophenylhydrazone (CCCP), which are mitochondrial oxidative phosphorylation uncouplers, exhibited anti-austerity activity against PANC-1 cells under glucose-starvation conditions [17]. Antimycin A, which inhibits the mitochondrial electron transport chain, reduces oxygen consumption in PANC-1 cells, whereas the uncouplers CCCP and secalonic acid D increase the oxygen consumption [17,61]. In other words, because an uncoupler blocks the phosphorylation step and activates the oxidation process, the oxygen consumption rate in the cells is increased. Conversely, the inhibitor of the mitochondrial electron transport chain prevents the oxidation process. Then, the oxygen consumption rate in the cells is decreased. Therefore, we further investigated the effects of compounds **1** and **2** on oxygen consumption in PANC-1 cells to confirm whether the compounds are uncouplers of oxidative phosphorylation in mitochondria or inhibitors of the mitochondrial electron transport chain. Compounds **1** and **2** were found to inhibit oxygen consumption to a similar extent as antimycin A (Figure 3). This result suggests that compounds **1** and **2** would inhibit the mitochondrial electron transport chain.

To confirm the functions of compounds **1** and **2**, the effects of the compounds on complexes I–V in the mitochondrial electron transport chain were examined using the Mito Check Complex Activity Assay Kit. Both compounds were found to inhibit the mitochondrial electron transport chain; however, the target complex of each compound differed (Table 2). Compound **2** selectively inhibited complex IV, with an IC_50_ value of 20 µM. In contrast, compound **1** was a broad inhibitor of the mitochondrial electron transport chain, with IC_50_ values of 90, 34, and 100 µM against complexes II, IV, and V, respectively. More specifically, 100 µM of compound **1** showed 9% of inhibition on complex III. One hundred micromolar of compound **2** exhibited 18%, 24%, and 6% of inhibition on complexes I, II, and III, respectively. Complex V was not inhibited by 100 µM of compound **2**. Thus, these compounds targeted the mitochondrial electron transport chain in PANC-1 cells; mitochondrial dysfunction in PANC-1 cells that adapted to glucose-deficient conditions led to growth inhibition. However, further studies are required to clarify the relationships between inhibition of the mitochondrial electron transport chain, anti-austerity activity, and selectivity of activity due to differences in culture conditions.

Activation of the Akt signaling pathway and endoplasmic reticulum (ER) stress response is important for the adaptation of cancer cells to nutrient-starvation conditions [62,63]. Some inhibitors of mitochondrial function are known to inhibit the activation of Akt signaling and/or the ER stress response (e.g., induction of GRP78 protein expression) [13,15,64]. Therefore, we strongly suggested that, similar to the effects noted for other inhibitors of mitochondrial function, inhibition of the mitochondrial electron transport chain by compounds **1** and **2** would disrupt Akt signaling and/or the ER stress response, ultimately inducing the death of PANC-1 cells that adapted to glucose-starvation conditions. A future plan for this confirmation will be done using Western blot.

## 3. Materials and Methods

### 3.1. General

Nuclear magnetic resonance ^1^H NMR (600 MHz), ^13^CNMR (150 MHz) spectra were obtained on a Varian-INOVA 600 instrument (Agilent Technologies, Inc., Santa Clara, CA, USA). Chemical shifts for ^1^H-NMR was reported in parts per million downfield from deuterochloroform as a reference standard. Coupling constants were in hertz (Hz). The following abbreviations are used for spin multiplicity: s = singlet, d = doublet, t = triplet, q = quartet, m = multiplet, and br = broad. Chemical shifts for ^13^C-NMR are reported in ppm relative to the center line of a triplet at 77.16 ppm for deuterochloroform. High-resolution mass spectra (HRMS) were obtained in positive electroscopy ionization (ESI), using leucine enkephalin as the internal standard. ESI-TOF-MS and MALDI-TOF-MS were performed on the Q-TOF Ultima API (Waters Co., Milford, MA, USA) and JMS-S3000 Spiral TOF (JEOL Ltd., Tokyo, Japan) instruments, respectively. Analytical thin-layer chromatography (TLC) was performed on the Merck precoated analytical plates, 0.25-mm-thick, silica gel 60 F_254_. Column chromatography separations were performed on KANTO CHEMICAL silica gel 60 N (spherical 63–210-mm mesh; Kanto Chemical Co., Inc. Osaka, Japan). Precoated TLC plates, 0.5-mm-thick, (Merck 60 F_254_; Merck KGaA, Darmstadt, Germany) were used for preparative TLC. HPLC was performed using a Hitachi L-6000 pump equipped with a Hitachi L-4000H UV detector (Hitachi High-Tech Science Corporation, Tokyo, Japan). Chemicals were purchased from Sigma-Aldrich (St. Louis, MO, USA) or Kishida Chemical Co., Ltd. (Osaka, Japan). Commercially available chemicals were used as purchased.

### 3.2. Materials

Dulbecco’s modified Eagle’s medium (DMEM), WST-8 colorimetric reagent, and KCN were purchased from Nacalai Tesque, Inc. (Kyoto, Japan). Fetal bovine serum (FBS) and dialyzed FBS were purchased from Equitech-Bio Inc. (Kerrville, TX, USA) and Thermo Fisher Scientific Inc. (Waltham, MA, USA), respectively. The Mito Check Complex Activity Assay Kit, used to evaluate the effect of test samples on mitochondrial complexes I–V, was obtained from Cayman Chemical (Ann Arbor, MI, USA). The oxygen utilization of cells and mitochondrial membrane potential were measured by using an Oxygen Consumption Rate Assay Kit from Cayman Chemical and JC-1 MitoMP Detection Kit from Dojindo Laboratories (Kumamoto, Japan), respectively. Rotenone, thenoyltrifluoroacetone (TTFA), carbonyl cyanide *m*-chlorophenylhydrazone (CCCP), antimycin A, and oligomycin mixture were purchased from Tokyo Chemical Industry Co. Ltd. (Tokyo, Japan), Wako Pure Chemical Industries, Ltd. (Osaka, Japan), Sigma-Aldrich (St. Louis, MO, USA), LKT Laboratories, Inc. (St. Paul, MN, USA), and Cayman Chemical, respectively.

### 3.3. Cell Culture and Bioassay

PANC-1 cells were maintained in DMEM supplemented with heat-inactivated 10% FBS and kanamycin (50 µg/mL) and grown at 37 °C in a 5% CO_2_ atmosphere. The nutrient-deprived PANC-1 cells were cultured in glucose-starved medium (basal medium (25 mM of N-2-hydroxyethylpiperazine-N-ethanesulfonic acid (HEPES) buffer (pH 7.4) containing 6.4-g/L NaCl, 700-mg/L NaHCO_3_, 400-mg/L KCl, 265-mg/L CaCl_2_·2H_2_O, 200-mg/L MgSO_4_·7H_2_O, 125-mg/L NaH_2_PO_4_, 0.1-mg/L Fe(NO_3_)·9H_2_O, 15-mg/L phenol red, 10 mL/L of Eagle′s minimum essential medium (MEM) vitamin solution (X100) (Gibco, Carlsbad, CA, USA), 200-mmol/L L-glutamine solution (Gibco), and 25-mg/L kanamycin) containing 10% dialyzed FBS). The general culture medium—that is, basal medium containing 10% FBS and 2.0 g/L glucose (final concentration, 25 mM)—was used for comparison. The assay was performed as previously described [14]. Briefly, PANC-1 cells (1 × 10^4^ cells/100 µL in 96-well plastic plates) were preincubated in DMEM containing 10% FBS for 24 h. The medium was then changed with either the general glucose medium or glucose-deficient medium to induce adaption to nutrient starvation. After 12-h incubation, serially diluted samples were added to the media. Then, the cells were incubated for an additional 12 h at 37 °C in a 5% CO_2_ atmosphere. Cell proliferation was identified using WST-8. The IC_50_ values were determined by linear interpolation of the growth inhibition curve. We assessed the selectivity of the antiproliferative activity (S.I.) on the basis of the difference in the IC_50_ values for the general culture and glucose-deficient media.

### 3.4. Evaluation of Mitochondrial Membrane Potential

The mitochondrial membrane potential of PANC-1 cells was evaluated using the JC-1 MitoMP Detection Kit (Dojindo Laboratories) following the manufacturer’s instructions. Briefly, PANC-1 cells (1.0 × 10^4^ cells/100 µL/well) that were adapted to the glucose-deficient medium in black/clear bottom 96-well plates (Corning Incorporated, Corning, NY, USA) were treated with the indicated concentrations of the compounds for 1 h. Subsequently, 100 µL of JC-1 working solution was added to each well, and the plate was incubated for 30 min. After 30-min incubation, the cells were washed with Hanks’ balanced salt solution (HBSS), and 100 µL of imaging buffer solution was added. The fluorescence intensity of the cells was assessed using an Infinite M1000 microplate reader (Tecan Group Ltd., Mannedorf, Switzerland) at Ex 485 nm and Em 535 nm (Green color fluorescence) and Ex 535 nm and Em 595 nm (Red color fluorescence). The ratio of the two fluorescence intensities (Red vs. Green) was then calculated.

### 3.5. Evaluation of Oxygen Consumption

The oxygen consumption of PANC-1 cells was analyzed using the Oxygen Consumption Rate Assay Kit (Cayman Chemical) following the manufacturer’s instructions. Briefly, precultured PANC-1 cells (8.0 × 10^4^ cells) in black/clear bottom 96-well plates (Corning Incorporated) were incubated for 12 h at 37 °C in general glucose medium. The medium was then replaced with 140 µL of fresh medium, and the test compound was added, followed by the addition of the phosphorescent probe to measure the oxygen consumption. Then, each well was sealed with 100 µL of mineral oil to prevent oxygen diffusion. The signals were measured using an Infinite M1000 microplate reader (Tecan Group Ltd., Zurich, Switzrland) in the time-resolved mode at Ex 380 nm and Em 650 nm for 180 min, with a 1-min time interval. Linear regression was applied after subtracting the blank, and the oxygen consumption rate was determined by the slope of each signal profile.

### 3.6. Measurement of Mitochondrial Complex Activity

The inhibitory activity of compound **1** on the mitochondrial electron transport chain (complexes I–V) was analyzed using MitoCheck Complex Activity Assay kits (Cayman Chemical) following the manufacturer’s instructions. The kits measured the enzymatic activity of each complex derived from bovine heart mitochondria in the following systems [65]: Mitochondrial complex I (NADH oxidase/coenzyme Q reductase) activity was determined by measuring the decrease in NADH oxidation, which is reflected by a decrease in absorbance at 340 nm. Mitochondrial complex II (succinate dehydrogenase/coenzyme Q reductase) activity was assessed on the basis of the reduction rate of 2, 6-dichlorophenolindophenol (protonated by reduced coenzyme Q), which is reflected by a decrease in absorbance at 600 nm. Mitochondrial complex III (coenzyme Q cytochrome *c* oxidoreductase) activity was determined on the basis of the cytochrome *c* reduction rate, which is reflected by an increase in absorbance at 550 nm. Mitochondrial complex IV (cytochrome *c* oxidase) activity was determined by measuring the cytochrome *c* oxidation rate, which is reflected by a decrease in absorbance at 550 nm. Mitochondrial complex V (F_1_F_0_ ATP synthase) activity was determined by the NADH oxidation rate, which can be monitored at 340 nm.

### 3.7. Isolation of Active Compounds ***1*** and ***2***

The marine-derived fungus 18B-15-3 was isolated from an unidentified marine sponge, which was collected on Pramuka Island, Indonesia in 2016. The strain was identified as *Aspergillus* sp. by Techno Suruga Laboratory Co., Ltd. (Shizuoka, Japan) on the basis of its morphological features and 5.8S rDNA sequence. *Aspergillus* sp. 18B-15-3 was cultured in rice medium (75-g unpolished rice and 150-mL artificial sea water) under static conditions at 30 °C for 15 days. The culture was extracted twice with acetone, and the organic solvents were evaporated under vacuum conditions to obtain a crude extract. The crude extract was partitioned into a water–EtOAc mixture (1:1).

For the bioassay, the active EtOAc fraction (5.2 g; IC_50_ = 25-μg/mL glucose-deprived medium and IC_50_ > 100-μg/mL general culture medium) was then partitioned into a *n*-hexane–90% aqueous MeOH mixture (1:1). The *n*-hexane fraction (2.3 g; IC_50_ = 15.0-μg/mL glucose-deficient medium and IC_50_ > 100-μg/mL general culture medium) was then fractionated using silica gel open column chromatography involving elution with a *n*-hexane–chloroform gradient to afford eight fractions (Fr. 1–Fr. 8). Among these fractions, Fr. 6 (165 mg; eluted by *n*-hexane-chloroform = 6:4) showed selective growth inhibition of the PANC-1 cells that were adapted to glucose starvation (IC_50_ = 2.0-μg/mL glucose-deficient medium and IC_50_ > 100-μg/mL general culture medium). Fr. 6 was then separated by normal-phase HPLC (COSMOSIL 5SL-II; 10-mm internal diameter × 250 mm; *n*-hexane-CHCl_3_ = 6:4) to afford three fractions (Fr. 6_1–Fr. 6_3). The active fraction 6_2 (69 mg; IC_50_ = 15-μg/mL glucose-starved medium and IC_50_ > 100-μg/mL general culture medium) was further purified by preparative TLC (running solvent: *n*-hexane-chloroform (4:6)) to obtain physcion (**1**, 4.0 mg) and 2-(2′,3-epoxy-1′,3′,5′-heptatrienyl)-6-hydroxy-5-(3-methyl-2-butenyl) benzaldehyde (**2**, 5.0 mg).

A formula of C_16_H_12_O_5_ was suggested for **1** on the basis of HR MALDI-TOF-MS observed at m/z of 285.0769 (M + H) ^+^, calculated 284.0763. ^1^H-NMR (600 MHz, CDCl_3_, δ_H_): 7.07 (1H, dd, *J* = 0.6, 1.8 Hz, H-2), 7.62 (1H, dd, *J* = 0.6, 1.8 Hz, H-4), 7.36 (1H, d, *J* = 2.4 Hz, H-5), 6.67 (1H, d, *J* = 2.4 Hz, H-7), 2.45 (3H, s, CH_3_-3), 3.93 (3H, s, OCH_3_-6), 12.11 (1H, s, OH-1), 12.31 (1H, s, OH-8); ^13^C-NMR (150 MHz, CDCl_3_, δc): 190.7 (C-9), 182.0 (C-10), 165.2 (C-8), 166.5 (C-6), 162.5 (C-1), 148.4 (C-3), 135.2 (C-10a), 133.2 (C-4a), 124.5 (C-2), 121.3 (C-4), 113.7 (C-9a), 110.2 (C-8a), 108.2 (C-5), 106.7 (C-7), 56.0 (6-OMe), 22.1 (3-Me) [23,24] (Figures S1–S5).

A formula of C_19_H_20_O_3_ was suggested for **2** on the basis of HR MALDI-TOF-MS observed at m/z of 297.1492 (M + H) ^+^, calculated 297.1491. ^1^H-NMR (600 MHz, CDCl_3_, δ_H_): 7.45 (1H, s, H-4), 10.25 (1H, s, H-7), 6.79 (1H, br s, H-1′), 6.32 (1H, d, *J* = 15.4 Hz, H-3′), 6.94 (1H, dd, *J* = 15.4, 10.8 Hz, H-4′), 6.23 (1H, m, H-5′), 5.98 (1H, dq, *J* = 14.9, 6.8 Hz, H-6′), 1.86 (3H, d, *J* = 6.4 Hz, H-7′), 3.41 (2H, d, *J* = 7.2 Hz, H-1′’), 5.33 (1H, m, H-2′’), 1.78 (3H, d, *J* = 0.8 Hz, H-4′’), 1.72 (3H, br s, H-5′’), 11.72 (1H, s, OH-6); ^13^C-NMR (150 MHz, CDCl_3_, δc): 193.0 (C-7), 157.7 (C-6, C-2′), 148.7 (C-3), 134.1 (C-3′’), 133.8 (C-6′), 132.5 (C-4′), 131.1 (C-5′), 128.7 (C-2), 126.7 (C-5), 121.2 (C-2′’), 119.3 (C-4), 116.9 (C-3′), 110.6 (C-1), 100.1 (C-1′), 27.6 (C-1′’), 25.8 (C-4′’), 18.6 (C-7′), 17.8 (C-5′’) [25,26] (Figures S6–S14).

### 3.8. Statistical Analysis

Data were shown in terms of mean  ±  standard error of three independent experiments. The differences between datasets were assessed by Dunnett’s test. Differences with *p*-values less than 0.05 were considered significant.

## 4. Conclusions

In our search for anti-austerity agents from marine medicinal resources, physcion (**1**) and 2-(2′,3-epoxy-1′,3′,5′-heptatrienyl)-6-hydroxy-5-(3-methyl-2-butenyl) benzaldehyde (**2**) were rediscovered from the marine-derived fungus *Aspergillus* sp. 18B-15-3 isolated from a marine sponge. In addition, our findings that the isolated compounds targeted the mitochondrial electron transport chain have not been previously reported. The anti-austerity activity of the compounds suggested that they inhibit the mitochondrial electron transport chain. To our knowledge, this is the first study to suggest that these compounds, which have anti-austerity activity, target the mitochondrial electron transport chain. The anti-austerity agents IACS-10759 (synthetic inhibitor of complex I) and arctigenin (originally isolated from *Arctium lappa*) are currently in clinical trials as anticancer drugs [18,19]. The current findings suggest that the compounds isolated in our study will also provide new options for cancer chemotherapy. However, further studies are required on the effects of these compounds on Akt signal transduction and the ER stress response in PANC-1 cells and their in vivo efficacy in a mouse xenograft model in order to develop these compounds as leads for drugs.

## Figures and Tables

**Table 1:** Cytotoxic activity of compounds **1** and **2** against human pancreatic carcinoma PANC-1 cells under glucose-starvation and general culture conditions.

Compounds		IC_50_ (µM)	
	Glucose − ^1^	Glucose + ^2^	S.I. ^3^
**1**	6.0 ± 0.1	1017 ± 0.05	169.5
**2**	1.7 ± 0.05	859 ± 1	505.3
Antimycin A ^4^	0.0003 ± 0.001	288 ± 0.5	960,000

^1^ Glucose-deficient medium, ^2^ general glucose medium, ^3^ selectivity index, and ^4^ positive control.

**Table 2:** Effects of compounds **1** and **2** on the mitochondrial electron transport chain.

			IC_50_ (µM)		
	Complex I	Complex II	Complex III	Complex IV	Complex V
**1**	100 ± 0.92	90 ± 0.11	>100 ± 4.43	34 ± 0.3	100 ± 0.92
**2**	>100 ± 1.60	>100 ± 0.92	>100 ± 3.36	20 ± 0.3	>100 ± 1.60
Positive control ^1^	0.19 ± 0.02	68 ± 0.01	0.03 ± 0.01	21 ± 0.0	0.19 µg/mL ± 0.03

^1^ Positive controls for complexes I, II, III, IV, and V were rotenone, thenoyltrifluoroacetone, antimycin A, KCN, and an oligomycin mixture, respectively.

## Supplementary Materials

The following are available online at https://www.mdpi.com/1660-3397/18/11/555/s1: Figure S1: HR-ESI-MS spectrum of compound **1**. Figure S2: ^1^H-NMR spectrum of compound **1**. Figure S3: Expanded ^1^H NMR spectrum of compound **1**. Figure S4: ^13^C NMR spectrum of compound **1.** Figure S5: Expanded ^13^C NMR spectrum of compound **1**. Figure S6: HR-ESI-MS spectrum of compound **2**. Figure S7: ^1^H-NMR spectrum of compound **2**. Figure S8: Expanded ^1^H NMR spectrum of compound **2** (1). Figure S9: Expanded ^1^H NMR spectrum of compound **2** (2). Figure S10: Expanded ^1^H NMR spectrum of compound **2** (3). Figure S11: ^13^C NMR spectrum of compound **2**. Figure S12: Expanded ^13^C NMR spectrum of compound **2** (1). Figure S13: Expanded ^13^C NMR spectrum of compound **2** (2). Figure S14: Expanded ^13^C NMR spectrum of compound **2** (3).
