# Supplementary material for: Mitochondrial Targeting in an Anti-Austerity Approach Involving Bioactive Metabolites Isolated from the Marine-Derived Fungus Aspergillus sp
Source: Mar Drugs. 2020 Nov 7;18(11):555. doi: 10.3390/md18110555 (PMC7694948; doi:10.3390/md18110555)
Supplement: Supplementary file 1 [file marinedrugs-18-00555-s001.pdf]

Supplementary Materials

Article

Mitochondrial Targeting in an Anti-austerity Approach involving Bioactive Metabolites Isolated from the Marine-Derived Fungus *Aspergillus* sp.

Waleed A Abdel-Naime [1,2] Atsushi Kimishima [1], Andi Setiawan [3], John Refaat Fahim [2], Mostafa A. Fouad [2], Mohamed Salah Kamel [2,4,*] and Masayoshi Arai [1,*]

[1] Graduate School of Pharmaceutical Sciences, Osaka University, 1-6 Yamadaoka, Suita, Osaka 565-0871, Japan; waleed_cognosy@yahoo.com (W.A.A.-N.); kimishima-a@phs.osaka-u.ac.jp (A.K.)

[2] Department of Pharmacognosy, Faculty of Pharmacy, Minia University, Minia 61519, Egypt; Johnrefaat82@yahoo.com (J.R.F.); m_fouad2000@yahoo.com (M.A.F.)

[3] Department of Chemistry, Faculty of Science, Lampung University, J1. Prof. Dr. Sumantri Brodjonegoro No. 1, Bandar Lampung 35145, Indonesia; andi.setiawan@fmipa.unila.ac.id

[4] Department of Pharmacognosy, Faculty of Pharmacy, Deraya University, Universities Zone, New Minia 61111, Egypt

* Correspondence: mskamel@mu.edu.eg (M.S.K.); araim@phs.osaka-u.ac.jp (M.A.); Tel: +20-86-211-0026 (M.S.K.); Fax: +20-86-211-0032 (M.S.K.); Tel./Fax: +81-66879-8215 (M.A.)

Abstract: The tumor microenvironment is a nutrient-deficient region that alters the cancer cell phenotype to aggravate cancer pathology. The ability of cancer cells to tolerate nutrient starvation is referred to as austerity. Compounds that preferentially target cancer cells growing under nutrient-deficient conditions are being employed in anti-austerity approaches in anticancer drug discovery. Therefore, in this study, we investigated physcion (**1**) and 2-(2',3-epoxy-1',3',5'-heptatrienyl)-6-hydroxy-5-(3-methyl-2-butenyl) benzaldehyde (**2**) obtained from a culture extract of the marine-derived fungus *Aspergillus* sp., which had been isolated from an unidentified marine sponge, as anti-austerity agents. The chemical structures of **1** and **2** were determined via spectroscopic analysis and comparison with authentic spectral data. Compounds **1** and **2** exhibited selective cytotoxicity against human pancreatic carcinoma PANC-1 cells cultured under glucose-deficient conditions, with IC_{50} values of 6.0 and 1.7 μM, respectively. Compound **2** showed higher selective growth-inhibitory activity (505-fold higher) under glucose-deficient conditions than under general culture conditions. Further analysis of the mechanism underlying the anti-austerity activity of compounds **1** and **2** against glucose-starved PANC-1 cells suggested that they inhibited the mitochondrial electron transport chain.

Keywords: marine-derived *Aspergillus* sp.; cancer; microenvironment; nutrient starvation; austerity; physcion; mitochondrial electron transport chain

Contents ………………………………………………………………………. page

**Figure S1:**	HR-ESI-MS spectrum of compound **1**………………………………	3
**Figure S2**:	^{1}H-NMR spectrum of compound **1** …………………………………	3
**Figure S3**:	Expanded ^{1}H NMR spectrum of compound **1** ……………………...	4
**Figure S4**	^{13}C NMR spectrum of compound **1** ………………………………...	4
**Figure S5**:	Expanded ^{13}C NMR spectrum of compound **1** ……………………..	5
**Figure S6**:	HR-ESI-MS spectrum of compound **2** ……………………………...	5
**Figure S7**:	^{1}H-NMR spectrum of compound **2** …………………………………	6
**Figure S8**:	Expanded ^{1}H NMR spectrum of compound **2** (1) …………………...	6
**Figure S9**:	Expanded ^{1}H NMR spectrum of compound **2** (2)……………….. …	7
**Figure S10**:	Expanded ^{1}H NMR spectrum of compound **2** (3) …………….. ….	7
**Figure S11**:	^{13}C NMR spectrum of compound **2** ………………………………...	8
**Figure S12**:	Expanded ^{13}C NMR spectrum of compound **2** (1)…………………..	8
**Figure S13**:	Expanded ^{13}C NMR spectrum of compound **2** (2)…………………..	9
**Figure S14**:	Expanded ^{13}C NMR spectrum of compound **2** (3)…………………..	9

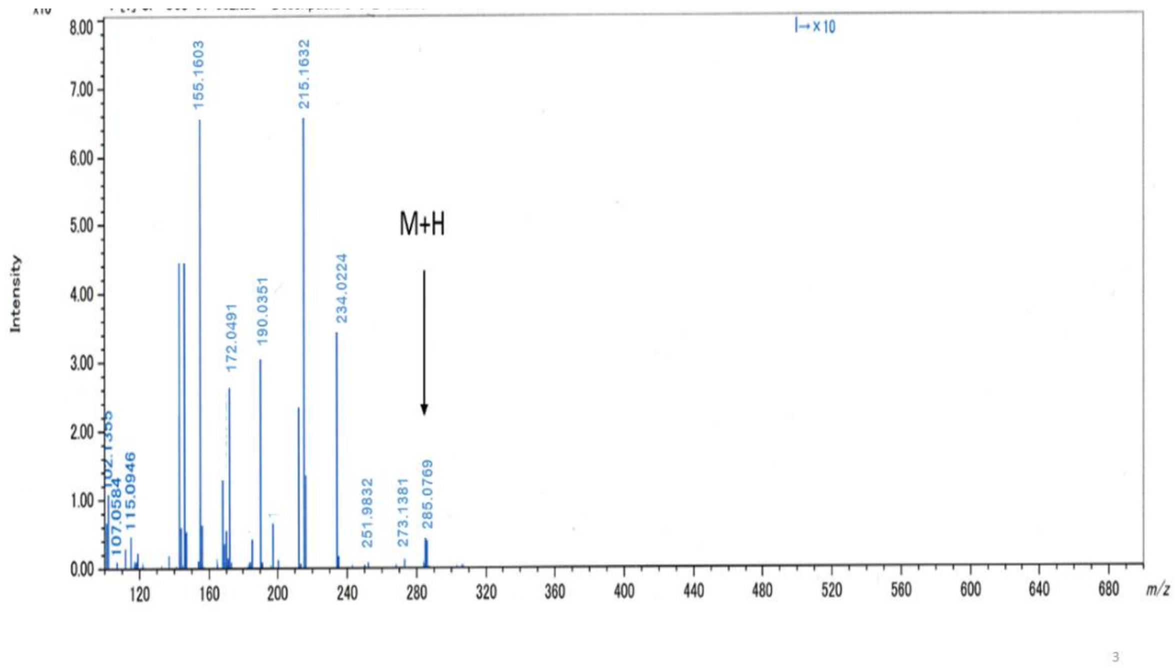

Figure S1: HR-MS (MALDI-TOF) spectrum of compound **1**

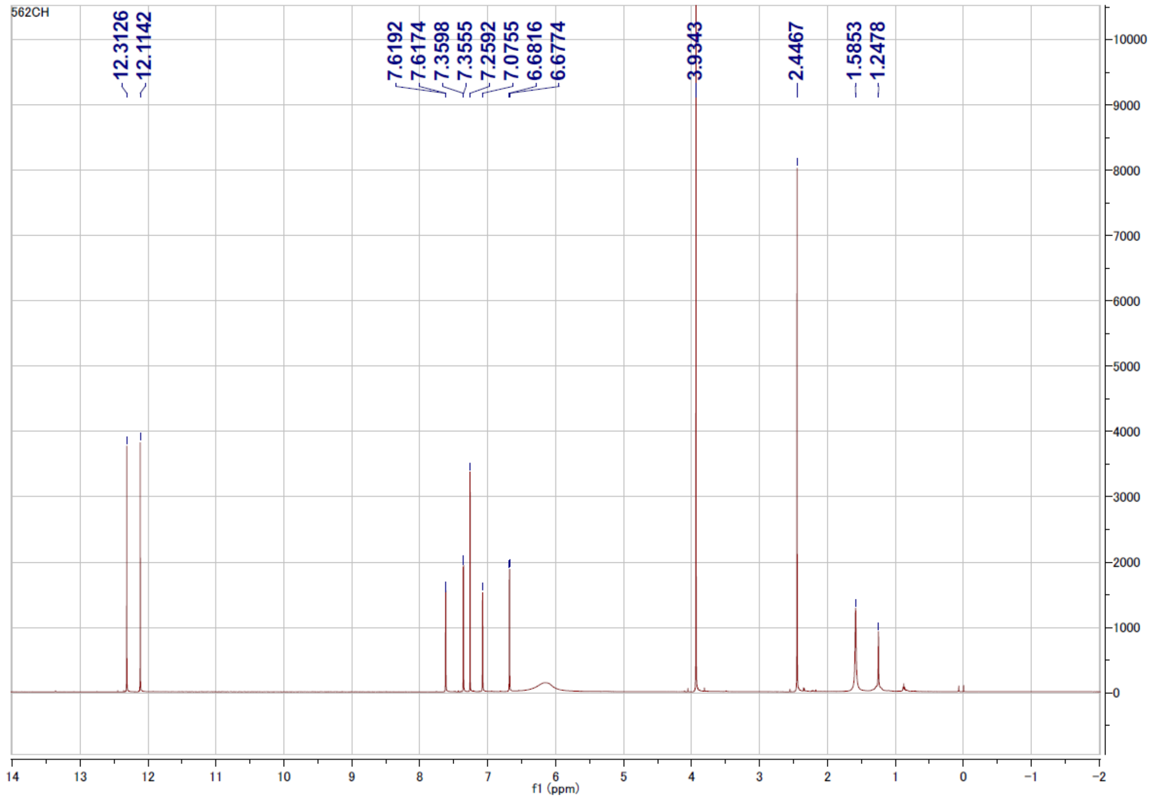

Figure S2: ^{1}H-NMR spectrum of compound **1**

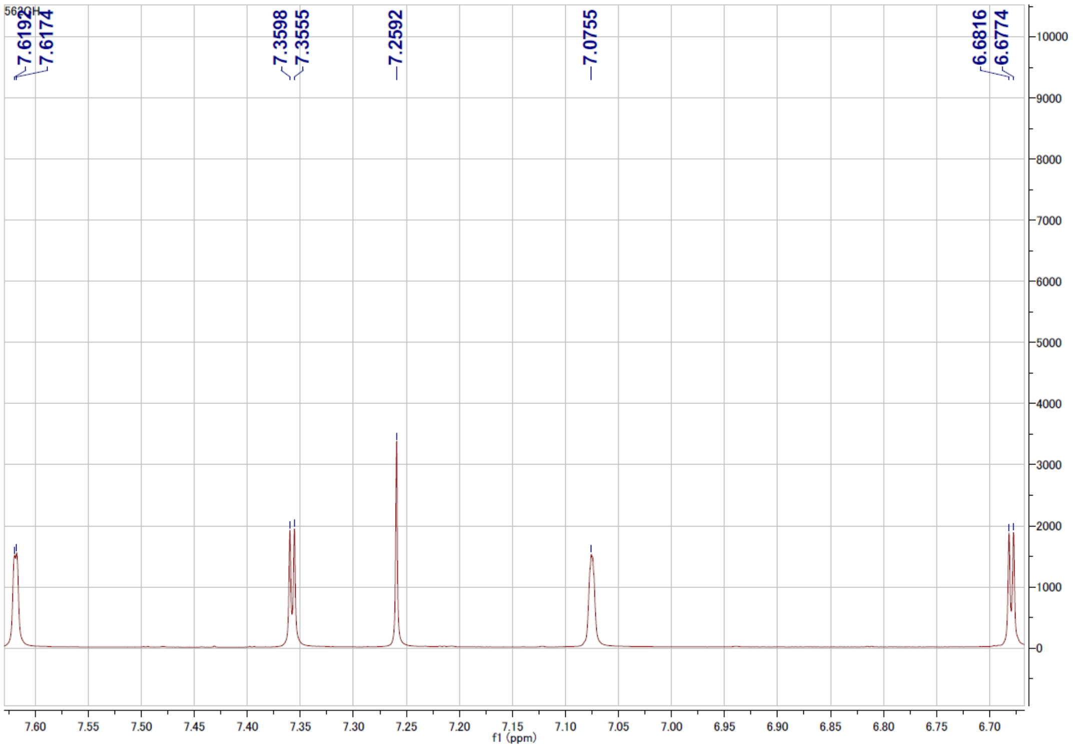

Figure S3: Expanded ^{1}H NMR spectrum of compound **1**

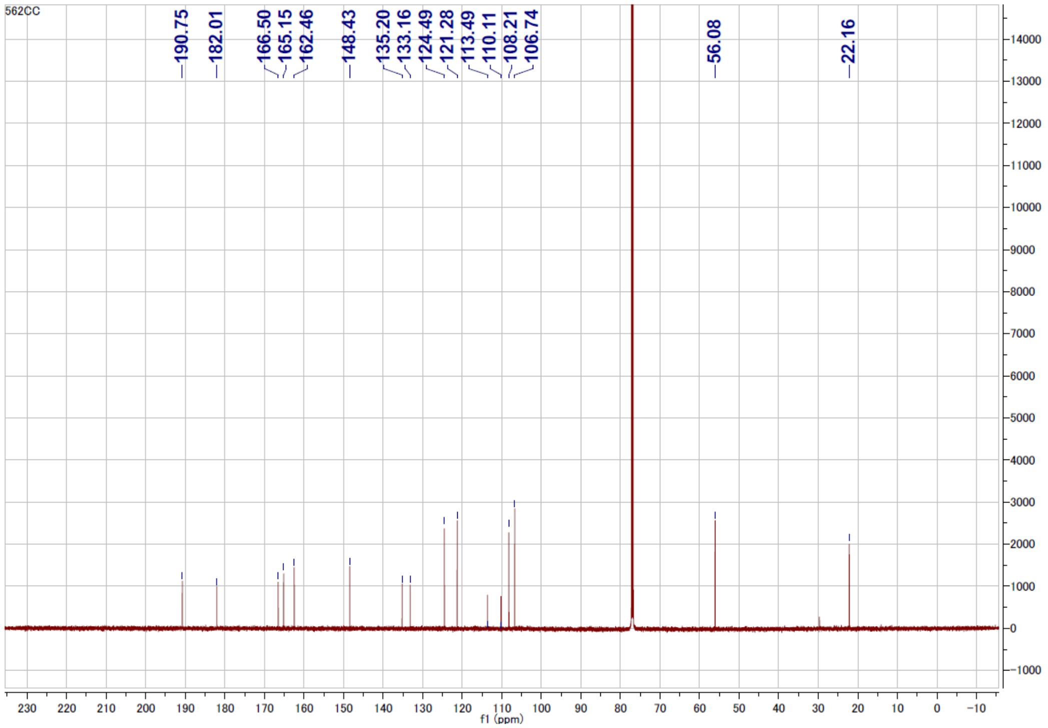

Figure S4: ^{13}C NMR spectrum of compound **1**

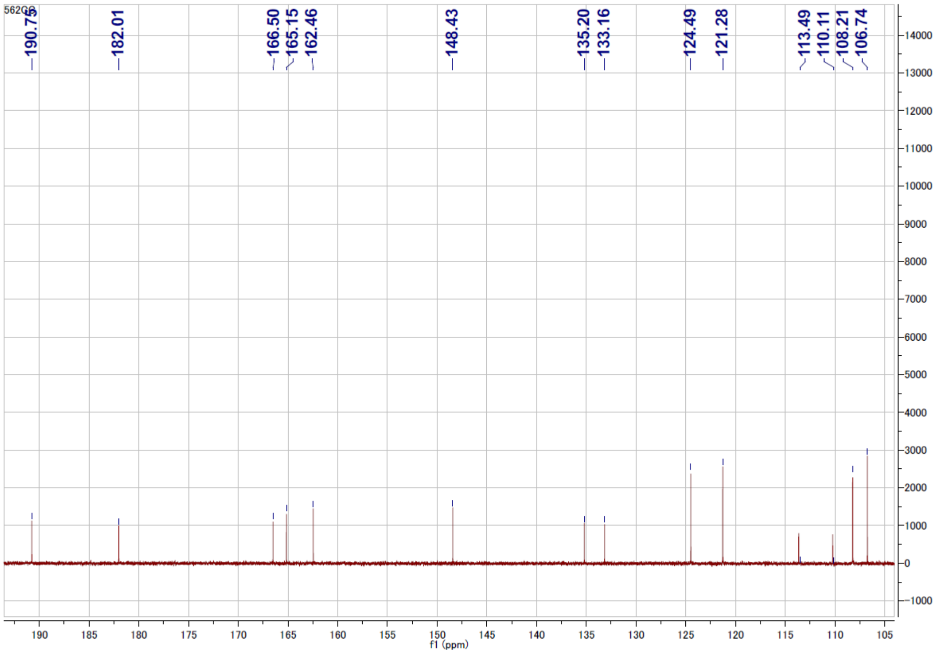

Figure S5: Expanded ^{13}C NMR spectrum of compound **1**

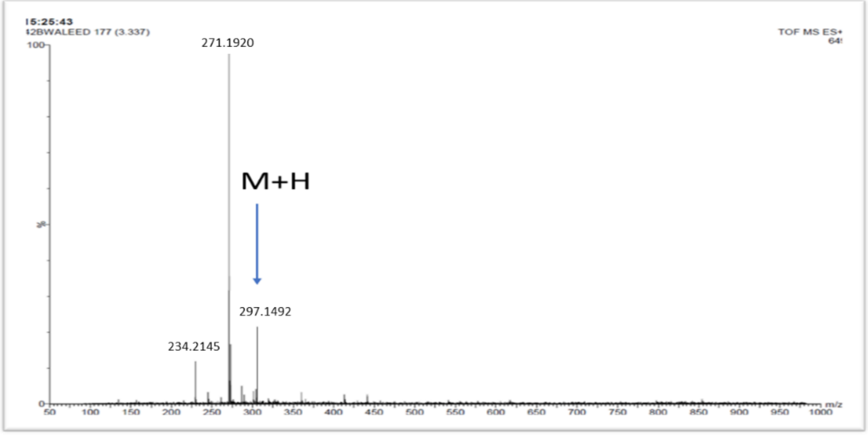

Figure S6: HR-ESI-MS spectrum of compound **2**

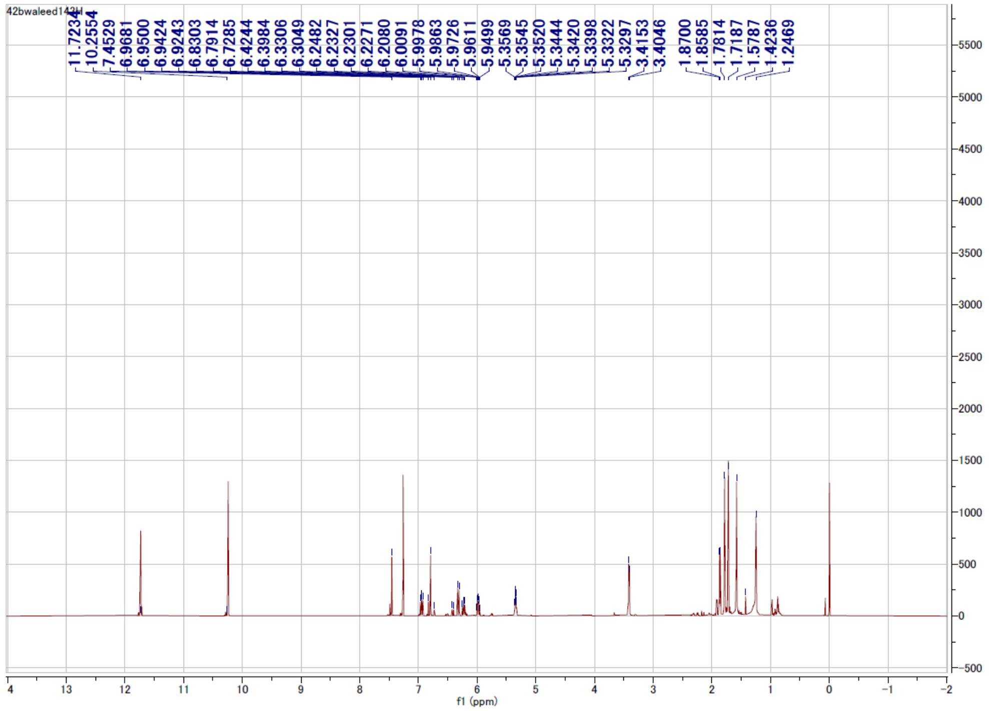

Figure S7: ^{1}H-NMR spectrum of compound **2**

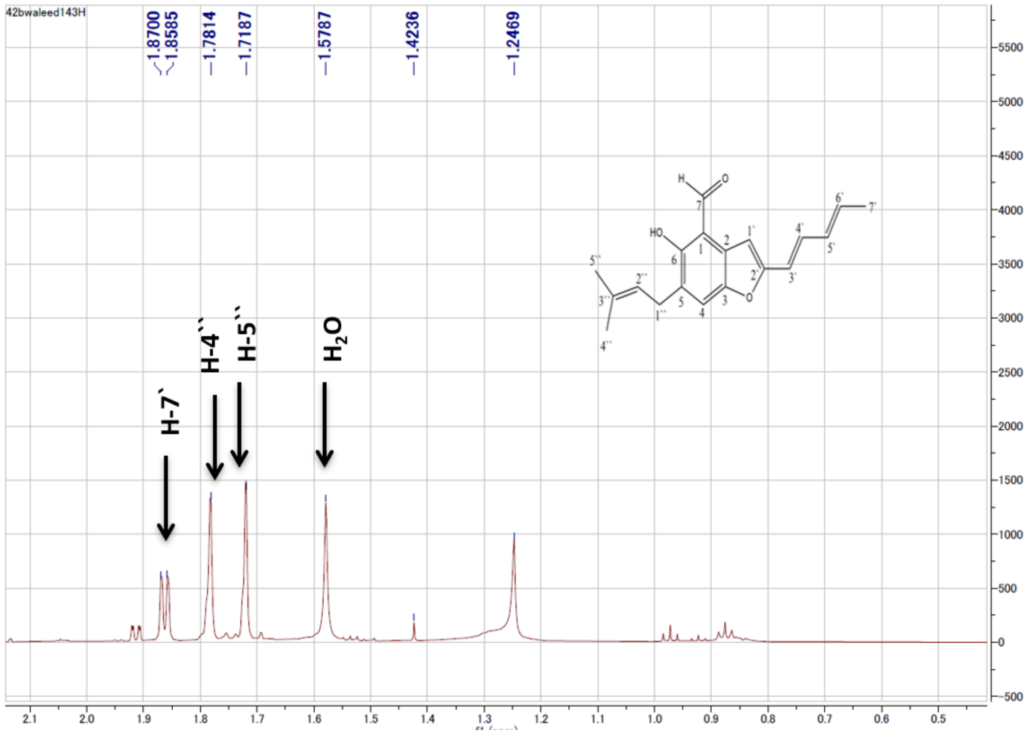

Figure S8: Expanded ^{1}H NMR spectrum of compound **2** (1)

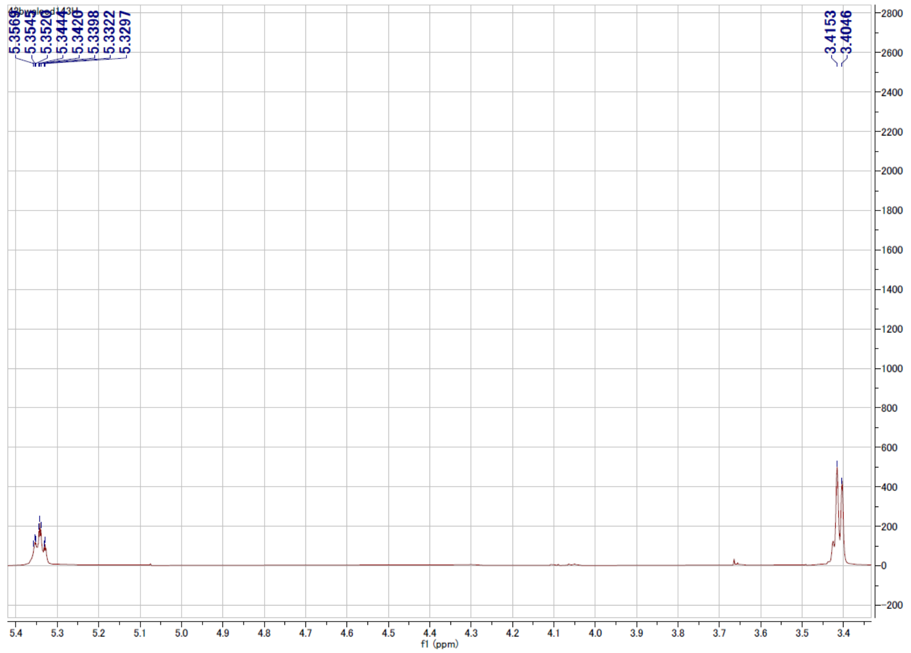

Figure S9: Expanded ^{1}H NMR spectrum of compound **2** (2)

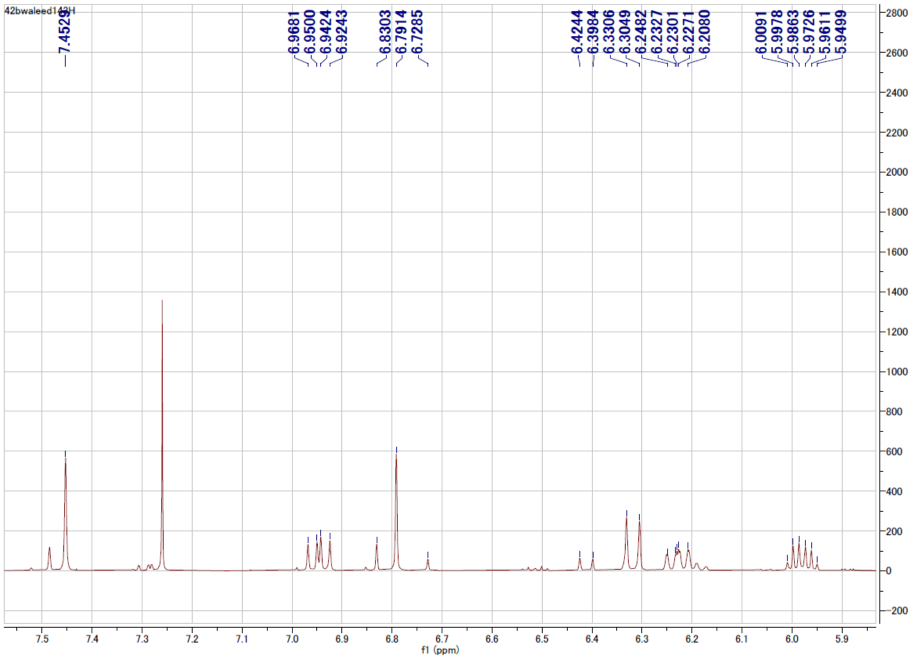

Figure S10: Expanded ^{1}H NMR spectrum of compound **2** (3)

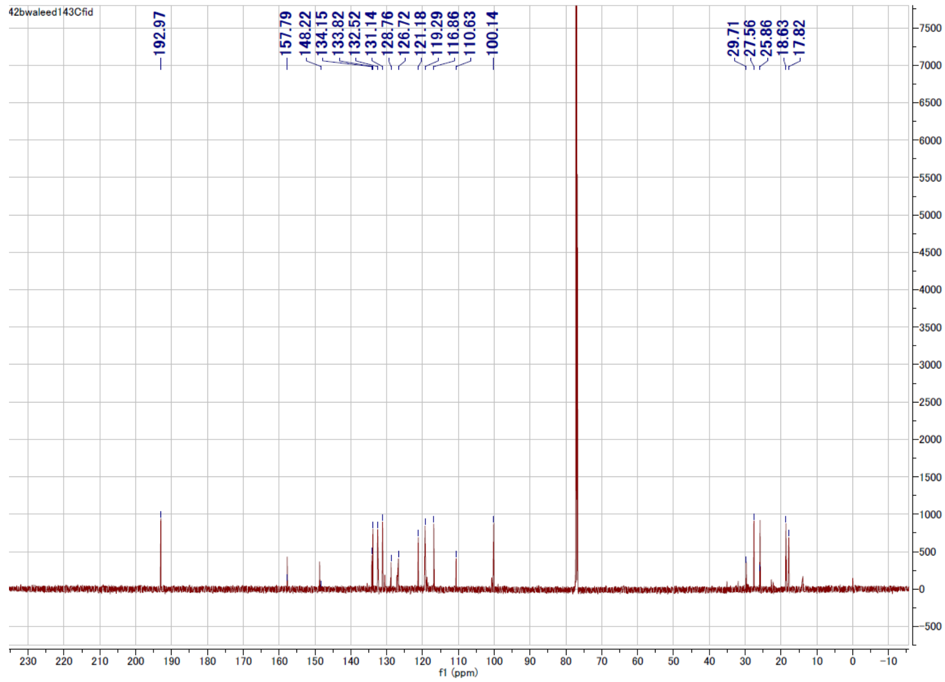

Figure S11: ^{13}C NMR spectrum of compound **2**

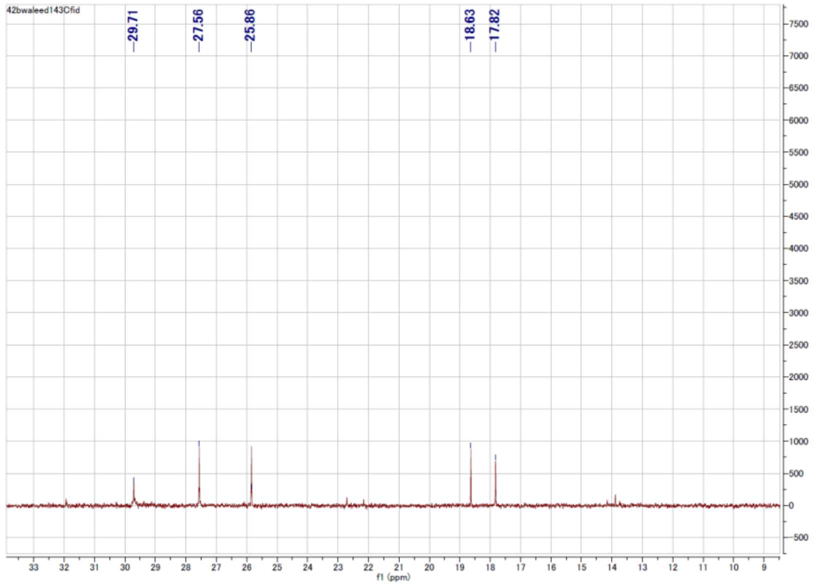

Figure S12: Expanded ^{13}C NMR spectrum of compound **2** (1)

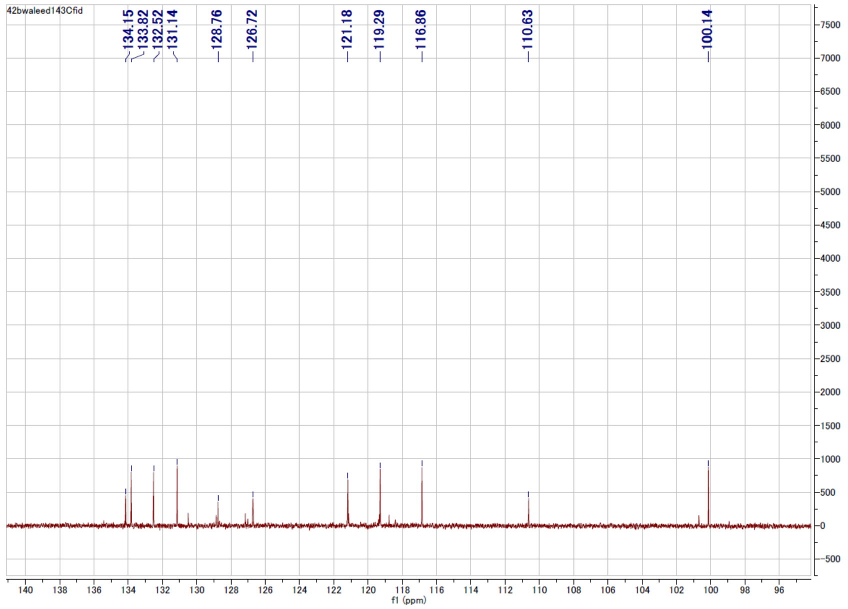

Figure S13: Expanded ^{13}C NMR spectrum of compound **2** (2)

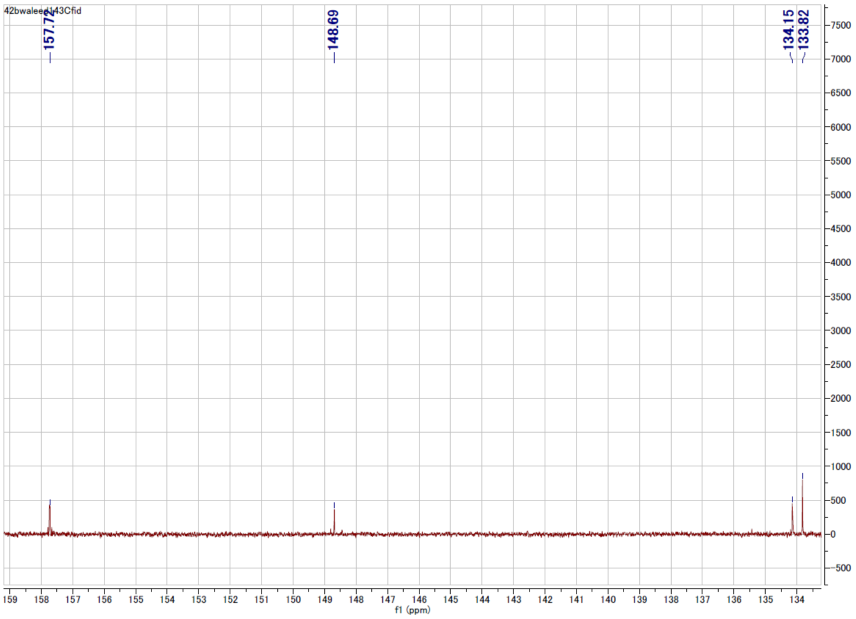

Figure S14: Expanded ^{13}C NMR spectrum of compound **2** (3)
